# Participant Perspectives: Inside the Rick Nishimura and Patrick T. O’Gara Emerging Faculty Leadership Academy

**DOI:** 10.1016/j.jaccas.2024.102489

**Published:** 2024-09-04

**Authors:** Onyedika J. Ilonze, Mustafa Husaini, Imo A. Ebong, Nupoor Narula, Joshua Levenson, Ran Lee, Jennifer A. Rymer, Monika Sanghavi, Louai Razzouk, Zain Ul Abideen Asad

**Affiliations:** a Division of Cardiovascular Medicine, Krannert Cardiovascular Research Center, Indiana University, Indianapolis, Indiana, USA; b Division of Cardiovascular Medicine, Washington University, St. Louis, Missouri, USA; c Division of Cardiology, University of California-Davis, Sacramento, California, USA; d Division of Cardiology, Weill Cornell Medicine, New York, New York, USA; e Division of Cardiovascular Medicine, UPMC, Pittsburgh, Pennsylvania, USA; f Division of Cardiovascular Medicine, Cleveland Clinic, Cleveland, Ohio, USA; g Department of Medicine, Duke University School of Medicine, Duke University, Durham, North Carolina, USA; h Division of Cardiology, University of Pennsylvania, Philadelphia, Pennsylvania, USA; i Cardiology Division, Department of Medicine, NYU Langone Health, NYU School of Medicine, New York, New York, USA; j Department of Medicine, University of Oklahoma Health Sciences Center, Oklahoma City, Oklahoma, USA

**Keywords:** emerging faculty, leadership, participant perspectives

Education is not the filling of a pail, but the lighting of a fire

—attributed to William Butler Yeats

## Background

Cardiovascular medicine faces complex and dynamic challenges that include a rapidly increasing body of knowledge, an aging patient population with multimorbidities, evolving health care policies, and the corporatization of health care.^1^^,^^2^ To address these challenges, a cardiologist must learn to navigate complex systems, inspire teams, drive patient care improvements, and educate peers. The skillset of teaching and strong leadership is vital for faculty leaders who play a pivotal role in shaping the future of cardiology. These individuals drive overarching cultural transformations, champion macrolevel shifts, influence microlevel improvements in communication, and foster a sense of belonging within the diverse membership of the American College of Cardiology (ACC). These early- to midcareer cardiologists, referred to as “emerging faculty leaders” are eager to learn from those already leading.

Cardiology-focused leadership programs across the country are sparse, with scant literature on their methodology or effectiveness. Education focused on effective adult learning, succinct communication strategies, and a culture of continuous improvement is key for leadership development. In recognition of this, the ACC has a core mission to enhance the education of practicing cardiologists through multiple leadership development programs. The Emerging Faculty Leadership Academy (EFLA)^3^ is a pivotal program that showcases the commitment of the ACC to leadership development. EFLA is a 3-day program aimed at teaching strategies for effective presentation and communication skills to emerging faculty leaders from a variety of backgrounds in cardiovascular medicine. This program aims to develop future leaders who will serve in a variety of roles as educators and leaders within their local institutions, regionally, and nationally in the ACC. This program facilitates mentorship from ACC leaders and provides opportunities for peer mentorship and networking.

This document represents the perspectives of the participants from the 2024 cohort of the EFLA, offering insights into the application process, 3-day workshop, and acquired skillsets. It further aims to spotlight the EFLA as a model for similar organizations that seek to foster emerging faculty to sustain a pipeline of diverse and talented leaders.

## The Application and Selection Process

**Figure 1:**
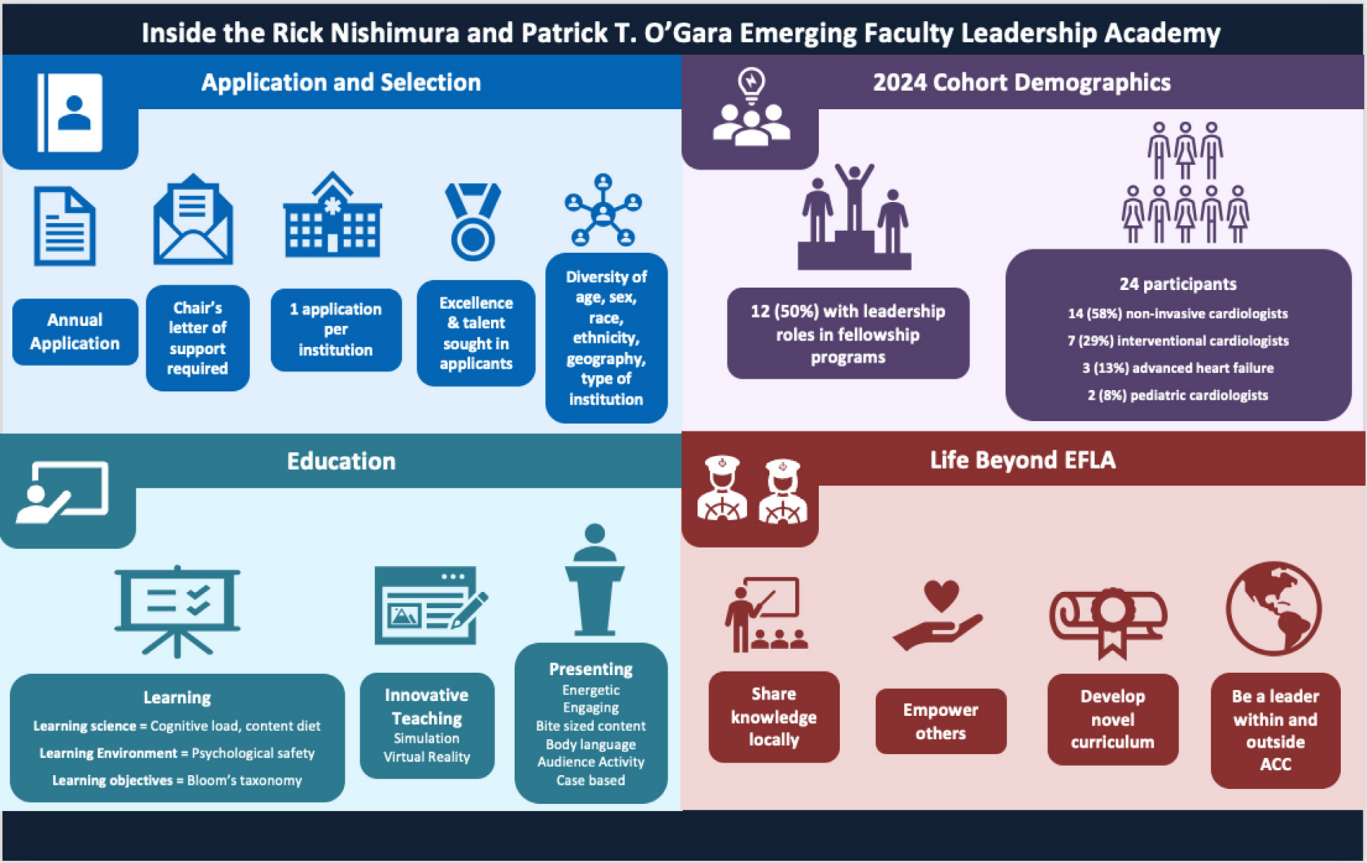
Visual Summary of Participant Perspectives Inside the ACC Emerging Faculty Leadership Academy.

The EFLA is a competitive program that accepts only 1 applicant per institution per year. The application and supporting letters need to show evidence of exceptional talent and commitment to teaching. A request for applications was posted on the ACC website, and potential applicants with an interest in education were encouraged to solicit nominations by their division chief. The selected EFLA cohort embodies inclusive excellence across age, sex, race/ethnicity, institution types (academic vs private), geographic locations, and subspecialty strata (Figure 1).

## The 2024 Cohort

Of the 24 participants in the 2024 cohort, two-thirds sought nominations from their institutional leadership, and the remaining were initially identified by their leadership. Most were first-time applicants, and 1 cohort member re-applied after seeking feedback and support from a prior EFLA graduate. Of the 24 attendees, 14 (58%) were noninvasive cardiologists with a variety of subspecialties, including sports medicine, vascular medicine, cardio-oncology, adult congenital heart disease, multimodality imaging, critical care, and clinical research; 7 (29%) were interventional cardiologists; and 3 (13%) were advanced heart failure, cardiomyopathies, and pulmonary hypertension specialists. Two attendees (8%) were pediatric cardiologists. In addition, 12 (50%) had leadership roles within their institution’s fellowship programs, and others had key programmatic leadership in cardio-oncology, women’s cardiovascular health, structural heart disease, sports cardiology, and hypertrophic cardiomyopathy. All attendees sought excellence in clinical care and education through effective means of presentation, communication, and leadership development.

## Teaching Skills Workshop: Education, Presentation Styles, and Leadership Lessons

Program faculty and speakers each year include the following: the course directors; the master physician educators; education and speaking specialists from within the College, and members of previous EFLA workshop classes (Acknowledgments). They embody the ethos of “Having light, Pass it on” as exemplified by many great educators before them.^4^

Before arrival, participants were grouped into cohorts of 4 to 5 individuals and tasked with identifying knowledge gaps within a thematic area of cardiology. On that basis, they were expected to record a 3-minute presentation and create a multiple-choice question with supporting rationale. Upon arrival at the ACC Heart House in Washington, DC, feedback on individual presentations was provided by the EFLA faculty. Participants subsequently revised their presentations and presented to the larger group in a 5-minute expanded form. The grouping enabled teamwork toward a common purpose. Feedback was also given on the multiple-choice question by group members and an experienced facilitator.

Didactic sessions included these areas of focus:

### Learning science

Areas of focus included: 1) maximizing sensory engagement to improve content retention; 2) reducing the learner’s cognitive load; and 3) exuding energy to positively influence the learners.

### Learning environment

An optimal learning environment strives to find the “sweet spot” between psychological safety and accountability. A framework for providing constructive feedback was discussed and included setting early expectations and having a genuine interest in the learner.

### Learning objectives

Objectives are a requirement for any educational activity based on continuing medical education. The critical nature of well-formed objectives was discussed and facilitated by the use of Bloom’s taxonomy. Each participant was required to create learning objectives as a roadmap to their presentation and was provided feedback on the quality of these objectives.

## Presentation and Moderator Skills

Effective presentation and moderator skills were taught and modeled by the course leadership, including habits and skillsets of highly effective presenters who use different methods to channel information succinctly to learners. Crafting high-yield slide decks with relevant content was implemented in small group feedback sessions. This ability to receive direct input on how to craft better visual and audio presentations was an essential part of the course’s impact on its participants. The course directors modeled their teaching by effectively engaging the audience with activities during presentations, recapping the day’s events, and chunking information into bite-sized content. An emphasis was placed on starting presentations with clinical cases and codifying learning objectives into key clinical questions to be answered. Effective moderation of panels by respecting the different panelists as well as engaging the audience in appropriate question-and-answer (Q&A) sessions was emphasized. During these lessons, the course directors modeled their teaching by effectively presenting, moderating, and leading an important agenda on how to be an effective presenter and ambassador of the ACC.

## Leadership

The importance of diversity, equity, and inclusion as a central pillar of the College was emphasized. This is important at our local institutions, on speaking panels or in manuscripts, and in all facets of cardiovascular medicine. Different leadership pathways for educators in the College, through local chapter engagement, state or national advocacy, and other faculty development programs were also reviewed. The EFLA faculty and alumni shared their leadership journeys within the ACC through expertly moderated Q&A sessions and fireside chats. The overall message was that EFLA is a springboard for future leadership opportunities within and outside the ACC.

Differences between the ACC EFLA and similar leadership programs offered locally or by other cardiology subspecialties include these: the EFLA: 1) is a shorter program consisting of online orientation and an intensive 3-day workshop, unlike other programs that last for 1 to 3 years; 2) focuses on producing excellent educators, unlike other programs that may have broader metrics of leadership; and 3) has diverse participation that facilitates networking across institutions and subspecialties, whereas subspecialty and local programs cannot achieve this aim.

## Life Beyond the ACC EFLA: Opportunities for Leadership in Cardiology

Independent of their practice setting, every cardiologist should be an effective educator because it is important to share information with patients, their family members, and colleagues.^5^ The EFLA has a pivotal role in addressing deficiencies in teaching competencies and developing cardiologists into effective educators. Skills acquired through EFLA can lead to increased participation in educational curricula development focused on effective content curation and delivery. EFLA participants are trained to be education leaders who are poised to lead novel educational practices that incorporate social networks, simulation technology, gamification, and virtual reality to improve learner satisfaction. The incorporation of social network platforms into learning activities may be useful because it is a highly effective learning management system that provides easy communication and can be assessed at the busy clinician’s convenience.^6^ The benefit of the EFLA program also extends beyond the local institution, inasmuch as newly created networks provide expanded opportunities for sustainable mentorship relationships and collaboration in various academic endeavors. EFLA participants are likely to become more committed to the College at the state or national level and to have a higher likelihood of being selected to serve as members of ACC committees or participate in other College activities.

The interaction between EFLA participants and faculty members provides an improved understanding of the mission and governance structure of the ACC and bolsters interactions with ACC staff. Because effective leadership is necessary for the delivery of high-quality education, research, and clinical practice,^7^ EFLA participants are encouraged to pursue further training related to their educational skills through advanced ACC educational and leadership training programs such as Advanced Educator Concepts (EFLA 2.0), the Mid-Career Women’s Leadership Development Program, the Leadership Academy, and the Clinical Trial Research Development Program. EFLA participants are also encouraged to pursue institutional and national leadership development opportunities. In furtherance of the educational mission of the ACC, EFLA graduates become equipped to advance educational initiatives locally and nationally, thereby meeting an existing need of “educators of the educators” among their peers.^3^ After completing the program, these faculty members can effectively serve as role models and resources for colleagues at their home institutions and throughout the ACC.^3^

## Conclusions

The EFLA is a testament to the ACC’s dedication to cultivating education leaders within cardiovascular medicine. The program equips participants with essential communication and presentation skills and fosters mentorship and networking. For the EFLA to remain relevant, there needs to be increased awareness of the program, particularly among early-career cardiologists. Finally, to grow and improve the program, course directors and ACC staff need to build a more robust infrastructure that allows tracking of graduate accomplishments, provides more sustained and constructive feedback to emerging educators, and enables measurement of outcomes that could demonstrate attainment of programmatic goals. In summary, the EFLA is a model for leadership development within medicine and underscores the need for similar initiatives.

## Funding Support and Author Disclosures

Dr Ilonze has received support from the PLUS program of Indiana University; and has received consulting fees from Bristol Myers Squibb and AstraZeneca. Dr Husaini has received support from Speakers Bureau of Bristol Myers Squibb and Amgen.
